# Total Neoadjuvant Approach for Borderline Resectable and Locally Advanced Pancreatic Adenocarcinoma—UK Tertiary Cancer Centre Experience

**DOI:** 10.3390/cancers18101597

**Published:** 2026-05-14

**Authors:** Kai Tai Derek Yeung, Simon Gomberg, William Hodgson, Petula Jefferies, David Cunningham, Sheela Rao, Ian Chau, Naureen Starling, Charlotte Fribbens, Avani Athauda, Diana Tait, Irene Chong, Arabella Hunt, Magnus T. Dillon, Sacheen Kumar, Long R. Jiao, Ricky H. Bhogal, Katharine Aitken

**Affiliations:** 1 Royal Marsden Hospital, London SW3 6JJ, UK; 2 The Institute of Cancer Research, London SW7 3RP, UK; 3 Surgery & Cancer, Imperial College London, London SW7 2AZ, UK

**Keywords:** pancreatic adenocarcinoma, neoadjuvant treatment, chemoradiation

## Abstract

**Introduction:** Pancreatic ductal adenocarcinoma (PDAC) remains a leading cause of cancer-related mortality. Radiological distinctions between borderline resectable (BR) and locally advanced disease (LA) are increasingly recognised as imperfect when considered without dynamic assessment. Neoadjuvant therapy (NAT) improves outcomes through tumour downstaging and early treatment of occult metastatic disease, but the optimal NAT strategy, particularly in BR disease, remains uncertain. Published data evaluating combined systemic anti-cancer therapies (SACT) with or without chemoradiation (CRT) are limited and heterogeneous. **Methods:** This is a single-centre retrospective analysis of 44 patients with BR PDAC and a comparator cohort of 121 patients with LA PDAC treated with a total neoadjuvant approach of SACT with or without CRT and surgical resection between June 2017 and September 2022. **Results:** Median overall survival (OS) did not differ significantly between BR and LA disease (18 vs. 16 months, *p* = 0.14). Following NAT, 47.7% of BR and 18.1% of LA patients were anatomically suitable for surgical resection. Among unresected BR and LA patients, those treated with CRT in addition to SACT had a median OS of 18 and 21 months respectively. In the resected subgroup, resection margin status was the primary factor associated with survival; with R0 resection conferring a substantial OS advantage over R1, irrespective of initial BR/LA classification as diagnosis (47 vs. 22 months, *p* < 0.001). **Conclusions:** Despite anatomical differences at diagnosis, BR and LA PDAC demonstrated comparable survival outcomes when treated with total neoadjuvant strategies in this cohort. These findings challenge traditional radiological staging-based treatment paradigms and confirm that a margin-negative surgical resection offered the greatest opportunity for long-term survival for BR/LA PDAC patients.

## Simple Summary

This single-centre retrospective study evaluated outcomes in patients with borderline resectable (BR) and locally advanced (LA) pancreatic ductal adenocarcinoma treated with total neoadjuvant therapy, including systemic chemotherapy with or without chemoradiotherapy, followed by possible surgical resection. Survival outcomes were similar between the BR and LA groups despite differences in anatomical staging at diagnosis. Nearly half of the BR patients and a smaller proportion of the LA patients became suitable for surgery after treatment. In unresected patients, the addition of chemoradiotherapy to systemic treatment was associated with encouraging survival outcomes. Among patients who underwent surgery, achieving a margin-negative (R0) resection was the strongest predictor of improved survival, regardless of initial disease classification.

## 1. Introduction

Pancreatic ductal adenocarcinoma (PDAC) is one of the leading causes of cancer-related mortality globally [1]. Its incidence continues to rise [2,3], particularly in younger women [4], whilst the 5-year survival has marginally improved from 2.4% to 7.2% over the last five decades in the United Kingdom. Non-metastatic PDAC can be categorised into three categories based on radiological appearances: resectable, borderline resectable (BR), locally advanced (LA) [5]. These categorisations have been used to help inform pre-operative decision-making about resectability and treatment sequencing. The incorporation of biological markers and patient performance status could result in a more comprehensive and clinically relevant categorisation in the future [6].

The overall survival (OS) benefit of adjuvant systemic anti-cancer therapy (SACT) is well established for resected PDAC [7,8,9], although real-world evidence suggests up to a third of patients are not able to start or complete a therapeutic course of adjuvant SACT [10]. Specifically in the United Kingdom, only 62% of patients receive any form of adjuvant treatment following Whipple’s procedure [11], which is similar to the PRODIGE 24 trial [8]. Neoadjuvant therapy (NAT) or a total neoadjuvant (TNT) approach, where all treatment is given prior to surgical resection, potentially offers several advantages: improved tolerability, downstaging to increase rates of resectability, disease control and eradication of micro metastatic disease, all of which may lead to an improvement in OS. It also has the benefit of sparing patients with biologically aggressive disease from futile major surgical resection if their PDAC progresses during NAT.

NAT has been associated with improved OS when compared to upfront resection in resectable [12] and BR PDAC [13,14] although the optimal NAT strategy for BR PDAC remains unclear [15]. Conflicting evidence exists, including the phase II NORPACT-1 trial, which did not demonstrate a survival benefit for neoadjuvant SACT in resectable PDAC [16]. The ESPAC5 [14] study demonstrated a survival advantage for NAT over upfront surgery but was not sufficiently powered to demonstrate which modality was superior. In BR PDAC, neoadjuvant chemoradiation (CRT) followed by surgery and adjuvant Gemcitabine demonstrated a survival advantage compared to upfront surgery followed by adjuvant Gemcitabine [17]. There is currently no established evidence supporting stereotactic body radiotherapy (SBRT) in BR PDAC [18] although further evaluation of its use is ongoing [19]. Finally, the recently published PREOPANC-2 trial reported no difference in OS between neoadjuvant Folinic acid, Fluorouracil, Irinotecan and Oxaliplatin (FOLFIRINOX) and Gemcitabine-based chemoradiotherapy for patients with resectable and BR PDAC [20].

In the UK, during the outbreak of the COVID-19 pandemic, the associated increased peri-operative risk and reduced surgical capacity led to more patients being treated with a greater number and longer duration of NATs. This included an increased use of additional cycles of neoadjuvant SACT and a national consensus-agreed hypofractionated CRT regimen following induction SACT for BR and LA PDAC [21]. To date, few published studies [22,23,24,25] report a TNT approach combining SACT +/− CRT, where all treatment is given prior to surgical resection in the treatment of BR and LA PDAC. The available data are limited by small sample sizes and significant heterogeneity in radiotherapy techniques, as well as SACT dosing and treatment regimens.

Here, we present our single-centre retrospective analysis of all patients with BR PDAC who have received total neoadjuvant treatment, with or without subsequent CRT and radical resection, alongside all patients with LA PDAC who serve as a comparator group. We acknowledge the inherent limitations in radiological differentiation between BR and LA disease; therefore, analysing both groups together allows a more pragmatic assessment of outcomes following the TNT approach across this spectrum of non-metastatic PDAC.

## 2. Methods

This retrospective study was approved by the Royal Marsden NHS Foundation Trust Service Evaluation Board (SE1414). It includes all patients who were diagnosed with BR or LA PDAC between June 2017 and September 2022, treated at the Royal Marsden Hospital. Patients were followed up until death or otherwise remained under active follow-up at the time of statistical analysis in December 2025. Except for four patients who did not have a recorded death and were lost to follow-up.

The brief treatment protocol is as follows: patients diagnosed with PDAC in our designated catchment area are referred to the Royal Marsden Hospital central multidisciplinary team meeting (MDM). All patients undergo staging with CT ± MRI and PET-CT. All patients had histological confirmation prior to commencing SACT. Serum tumour markers are measured to facilitate assessment of response to treatment.

Patients were treated with 12 cycles of neoadjuvant FOLFIRINOX unless contraindicated by comorbidity, toxicity, or allergy. Radiological reassessment was performed mid-treatment and on completion of SACT. Cases were then re-discussed at the MDM with reference to radiological findings and tumour marker trends. Patients with radiologically threatened margins involvement (potential R1 resection) are then considered for chemoradiotherapy (CRT). Patients who were not down-staged adequately to enable trial of surgical resection, decline or are unfit for surgery are treated with definitive doses of CRT or SBRT, although during the time period of this study, SBRT was not routinely available. Patients who developed metastatic disease during NAT were not offered CRT. Surgical trial dissection +/− resection is offered 6–8 weeks after completing NAT to those demonstrating radiological likelihood of successful margin-negative resection, sustained fall in Ca19-9 (in secretors), and a sustained metabolic response on PET. There are no strict Ca19-9 criteria.

Radiotherapy was planned on a contrast CT scan with 4D CT for motion management. The tumour and involved nodes were targeted without elective nodal irradiation. The dose fractionation delivered was 50.4–54 Gy/28–30# prior to the COVID-19 pandemic. From 2020, this was altered to a hypofractionated regimen of 45 Gy/15#, or 40 Gy/15# if there were concerns over risk of increased toxicity, with the option of 36 Gy/15# for borderline resectable disease. Radiotherapy was given with concurrent Capecitabine 1600 mg/m^2^ in two divided doses given on days of radiotherapy only.

The primary outcome of the study was to assess the difference in OS between BR and LA PDAC groups in this cohort. Secondary outcomes include the assessment of the potential therapeutic effect of chemoradiation and factors that influence OS between groups. OS was calculated from the first day of SACT to allow meaningful comparison between non-surgical and surgical groups.

### Statistical Analysis

All statistical analysis and graphics were produced using RStudio (Posit software, Boston, MA, USA, Version 2025.05.1+513). Categorical data are presented with absolute numbers and percentages. Continuous data are presented as medians with interquartile range. Kaplan–Meier curves were utilised to compare and analyse OS. Univariate and Multivariate Cox Proportional Analyses were performed where appropriate. Missing data fields, which were minimal in this database, were omitted during analysis. The log-rank test was used to compare study groups. Statistical significance was defined by *p* ≤ 0.05. Power calculation was not undertaken due to the retrospective nature of the study.

## 3. Results

**Figure 1:**
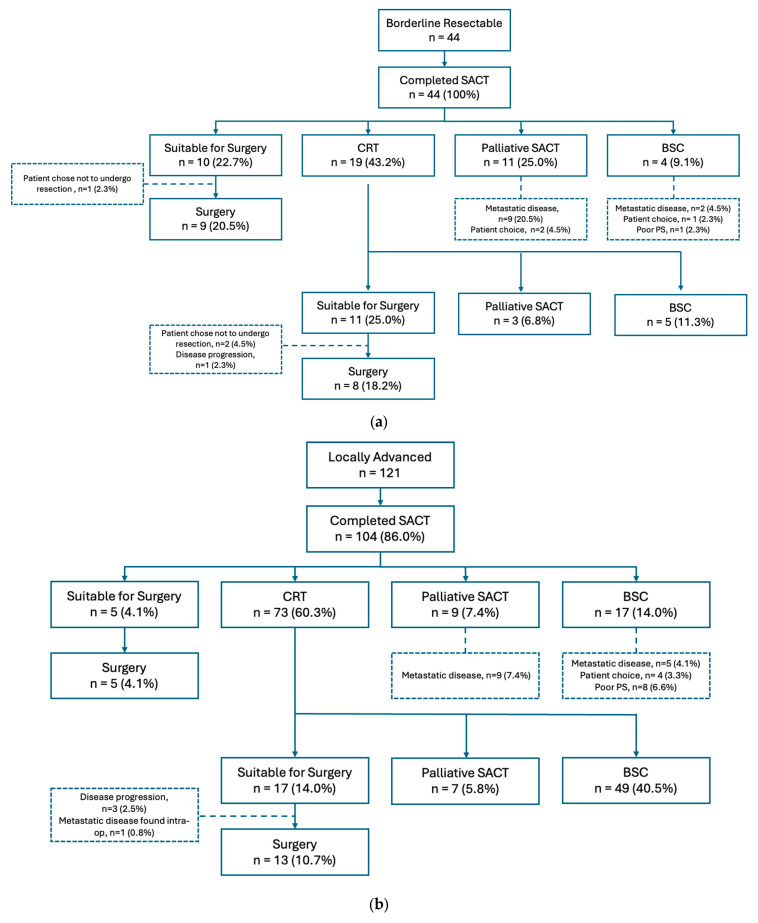
(**a**). Flow diagram demonstrating treatment pathway and outcomes of patients with borderline resectable disease. PS = performance status. (**b**) Flow diagram demonstrating treatment pathway and outcomes of patients with locally advanced disease. PS = performance status.

A total of 44 BR patients were compared to 121 LA patients in this retrospective cohort study. There were no significant differences in Age, BMI, ECOG Performance status and baseline median serum Ca19-9 levels. As expected, BR patients had a significantly lower burden of T4 disease at the point of diagnosis (Table 1, *p* < 0.001). FOLFIRINOX was the most common SACT regimen, followed by Gemcitabine/Capecitabine in both groups. Figure 1a,b illustrates the outcomes of patients following neoadjuvant SACT +/− CRT. Following neoadjuvant treatment, 47.7% of BR and 18.1% of LA patients were anatomically suitable for trial of surgical dissection. Eventually, 38.7% of BR and 14.8% of LA patients underwent successful resection.

**Figure 2:**
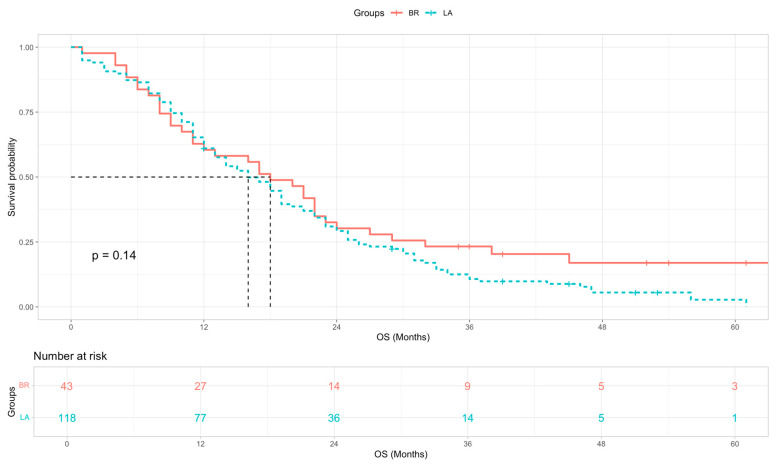
Kaplan–Meier OS curves for BR (red) and LA (blue) PDAC patients. Univariate Cox HR = 1.33 (95% CI 0.90–1.95, *p* = 0.148). Survival was compared using the log-rank test (χ^2^ = 2.19, *p* = 0.14).

**Figure 3:**
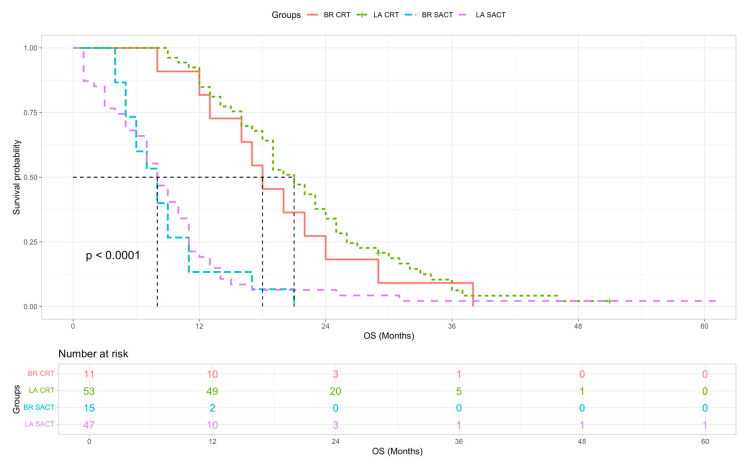
Kaplan–Meier OS curves for patients who did not undergo surgical resection—BR CRT (red), LA CRT (green), BR SACT (blue) and LA SACT (purple). Univariate Cox HR derived with LA SACT as reference group: BR CRT HR = 0.34 (95% CI 0.17–0.66, *p* = 0.002); LA CRT HR = 0.28 (95% CI 0.18–0.42, *p* < 0.001); BR SACT HR = 1.35 (95% CI 0.75–2.44, *p* = 0.322). Survival was compared using a multi-group log-rank test (χ^2^ = 55.5, df = 3, *p* < 0.0001).

The median survival of all BR and LA patients in this study was not significantly different at 18 vs. 16 months respectively (Figure 2, log-rank χ^2^ = 2.19, *p* = 0.14 and Cox HR = 1.33 (95% CI 0.90–1.95, *p* = 0.148). In patients who did not undergo surgical resection, both BR and LA patients who only received SACT had a median OS of 8 months. The main reason these patients did not receive CRT was the development of metastatic disease. BR and LA patients who were suitable and treated with further CRT had a median OS of 18 and 21 months respectively (Figure 3).

On univariate analysis, performance status, initial Ca19-9, N2 disease at diagnosis, treatment with FOLFIRINOX, as well as treatment with CRT and surgery were associated with OS (Table 2). On multivariate analysis, only treatment with CRT HR = 0.27 (95% CI 0.18–0.40), *p* < 0.001 and surgery HR = 0.15 (95% CI 0.09–0.27), *p* < 0.001 remained independent predictors of better OS.

Patients who underwent resection were further explored as a subgroup. BR and LA cohorts were similar with respect to age, BMI, sex distribution, ECOG performance status, and CA19-9 at diagnosis. Neoadjuvant SACT strategies were comparable, with 94.1% BR and 83.3% LA patients receiving FOLFIRINOX with similar rates of treatment completion and grade 3 SACT toxicity (see Table 3). As expected, tumour stage at diagnosis differed significantly between groups, with BR patients having T2–T3 disease and LA patients predominantly T4 (Table 3, *p* < 0.001). LA patients also demonstrated a higher nodal burden (Table 3, *p* = 0.008). Despite these differences, the operative approach and complexity were similar, including comparable rates of venous resection and R1 resection. Postoperative histological staging, morbidity, including pancreatic fistula, delayed gastric emptying, major complications (Clavien–Dindo ≥ 3) and 30-day readmissions did not differ significantly between groups. There were also no significant differences in postoperative morbidity and 30-day readmissions when comparing patients who were treated with neoadjuvant SACT to patients treated with neoadjuvant SACT and CRT (Supplementary Table S1). There were no type C postoperative pancreatic fistulas and no 90-day mortalities within the surgical subgroup.

**Figure 4:**
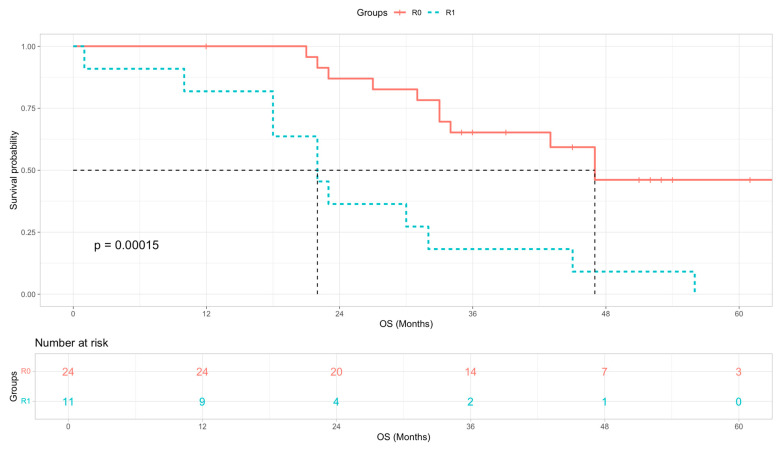
Kaplan–Meier OS curves for R0 (red) and R1 (blue) resected PDAC patients. Univariate Cox HR = 4.50 (95% CI 01.91–10.61, *p* < 0.001). Survival was compared using the log-rank test (χ^2^ = 14.34, *p* = 0.00015).

In this subgroup, survival did not differ significantly between resected patients with BR- and LA-disease, with median OS of 45 and 34 months respectively (Table 4, log-rank χ^2^ = 0.55, *p* = 0.458 and Cox HR = 1.37 (95% CI 0.58–1.37, *p* = 0.467)). Similarly, no statistically significant difference in survival was observed between patients treated with neoadjuvant SACT and CRT versus neoadjuvant SACT alone, despite a numerically longer median survival in the CRT group (33 vs. 22 months; Table 4, log-rank χ^2^ = 1.89, *p* = 0.168 and Cox HR = 0.56 (95% CI 0.24–1.31, *p* = 0.181)). In contrast, resection margin status was strongly associated with OS. Patients achieving an R0 resection had a significantly longer median OS compared with those with R1 resections, 47 vs. 22 months. (Figure 4, log-rank χ^2^ = 14.34, *p* = 0.00015 and Cox HR = 4.50 (95% CI 1.91–10.61, *p* < 0.001)). Multivariate analysis in this subgroup was not performed as only 22 out of 35 patients had passed away at the time of analysis. Resected R1 patients were also compared to non-surgery patients treated with SACT and CRT. The respective median OS were 22 vs. 20 months (Table 4, log-rank χ^2^ = 1.12, *p* = 0.289 and Cox HR = 0.70 (95% CI 0.36–1.38, *p* = 0.306)).

Recurrence rates were similar between BR and LA patients who underwent resection, occurring in 64.7% and 66.7% of patients respectively. Median time to recurrence did not significantly differ between groups (9 months in both cohorts, Table 5, *p* = 0.692), nor did the proportion of patients receiving palliative treatment following recurrence. R1 patients had a higher rate of recurrence but this was not statistically significant (Table 5, 90.8% vs. 54.1, *p* = 0.055). However, the pattern of recurrence differed significantly, with LA patients more likely to develop isolated locoregional recurrence (Table 5, 41.7% vs. 0%, *p* = 0.031), whereas BR patients more frequently developed combined locoregional and distant recurrence. When stratified by treatment modality, recurrence occurred more frequently in patients who received neoadjuvant SACT only, although this difference did not reach statistical significance (85.7% vs. 52.4%; Table 5, *p* = 0.095). Distribution of recurrence (locoregional, distant, or combined), time to recurrence, and subsequent palliative treatment were similar between SACT and CRT groups (see Table 5).

## 4. Discussion

In this study, we compared outcomes between BR- and LA-PDAC patients treated with a total neoadjuvant approach. We acknowledge the difference in patient numbers between BR/LA cohorts. However, the characteristics of BR and LA patients were otherwise well matched at baseline. LA patients had a substantially higher burden of T4 disease at diagnosis; however, the two groups were not associated with a difference in OS either in the full cohort or among those who ultimately underwent surgical resection. Postoperative histological staging was also not significantly different between BR and LA cases. These findings challenge the traditional dichotomisation of radiologically defined BR and LA disease as prognostically distinct entities and suggest that tumour biology and treatment response may be more relevant determinants of outcome than initial anatomical staging alone [26]. Moreover, BR patients are often grouped with upfront resectable disease in the literature, yet our data suggest their biology and prognosis may more closely resemble that of LA disease. Following NAT, 47.7% of BR and 18.1% of LA patients were anatomically suitable for trial of surgical dissection, which is consistent with other contemporary series [27,28]. BR patients were more likely to be anatomically suitable for surgical exploration and ultimately undergo resection compared with LA patients. Within the BR group, three (7%) of patients chose not to undergo major surgery, contributing to a lower rate of eventual resection at 38.6%.

There is a growing body of evidence supporting a NAT in BR and LA pancreatic cancer. Both SACT choices [29] and duration [30] appear to be important. In this study, the majority of patients who underwent resection received a median of ten to eleven cycles of FOLFIRINOX, in keeping with prior studies demonstrating superior response and resection rates compared with Gemcitabine-based regimens [27]. Although treatment-related toxicity remains a concern—grade 3 toxicity occurred in 48–53% of patients treated with FOLFIRINOX—yet more than half of patients were able to complete the intended SACT course, supporting the feasibility of TNT in selected patients. Notably, many published trials have incorporated shorter neoadjuvant regimens. The Alliance A021501 trial treated patients with seven to eight cycles of neoadjuvant FOLFIRINOX [31], as did the PREOPANC-2 trial [17] with eight cycles of neoadjuvant FOLFIRINOX, and in ESPAC 5, only four cycles in the FOLFIRINOX arm [14]. A recent meta-analysis demonstrated that the therapeutic efficacy of neoadjuvant CRT becomes significant only in patients treated with five or more cycles of induction SACT [32], therefore strengthening the rationale for a TNT approach.

Within this study, resected BR and LA patients remained comparable across peri-operative and oncological metrics. Rates of venous reconstruction, margin positivity, postoperative morbidity, and readmission were similar between groups. Following CRT, surgical resection may be technically challenging due to extensive oedema, desmoplastic reaction and fibrosis. In our experience, the optimal time for surgery is 6–8 weeks following completion of CRT. CRT patients who underwent resection did not experience excess operative risk or inferior postoperative outcomes, reinforcing the safety and feasibility of surgery in line with reported trials involving neoadjuvant radiotherapy [23], including the Alliance A021501 trial [33].

Importantly, median OS following resection did not differ significantly between BR and LA disease, underscoring that once resection is achieved, initial resectability status has limited prognostic value. In patients who did undergo surgical resection, margin status was still the most powerful determinant of survival. Patients with R0 resections had more than double the median OS compared with R1 resections (47 vs. 22 months), regardless of whether BR or LA at baseline. These findings highlight the importance of achieving clear margins, although R0 rates were not statistically different between groups in this study. Reliable preoperative prediction of margin status remains a challenge. In our cohort, R1-resected patients achieved a median OS that was indistinguishable from non-surgical patients treated with SACT and CRT (22 vs. 20 months). Acknowledging the inherent selection bias of a retrospective comparison, this finding suggests that patients destined for an R1 resection may gain very limited additional benefit from surgery and underscores the need for improved preoperative tools for margin prediction.

The CONKO 007 trial used modern intensity modulated radiotherapy and SACT regimens, FOLFIRINOX or Gemcitabine. It had similar resection rates of 35.5 to 36.5% in both arms and demonstrated increased R0 resection rates and complete pathological response in the chemotherapy-chemoradiation arm. Negative resection margins were associated with improved OS but this did not translate to an OS benefit for the entire cohort [34]. However, a recent meta-analysis of over 1500 patients reported neoadjuvant CRT to be associated with significantly improved R0 rates [32], highlighting its potential role in optimising local disease control. When stratified by treatment modality, recurrence following surgery was numerically lower in patients who received CRT compared with SACT alone (52% vs. 85%, *p* = 0.095), this did not reach statistical significance, possibly due to limited sample size.

Among patients who did not proceed to surgery, the addition of CRT following SACT led to meaningful OS and was the only factor to impact OS other than surgery following multivariate analysis. While this analysis is non-randomised and subject to treatment selection bias, the observed association with survival suggests that CRT may confer a meaningful benefit in patients with non-resected disease, potentially through improved locoregional disease control. Multimodality therapy, combining SACT with subsequent radiotherapy, has been studied in LA PDAC with varying results. The LAP-07 study assessed the addition of CRT following Gemcitabine +/− Erlotinib and found a progression-free survival benefit but no OS benefit [35].

The contribution of CRT in the treatment of BR PDAC remains an area of debate [36] but amongst resected BR and LA patients, those who received CRT in addition to SACT had numerically longer survival than those who proceeded to surgery after SACT alone (33 months vs. 22 months, *p* = 0.168). The authors hypothesise a potential synergistic effect of CRT in optimising surgical candidates, possibly by enhancing margin negativity and controlling microlocal disease [37]. The mechanisms driving this clinical benefit require further investigation but may stem from CRT’s effect on PDAC’s immunosuppressive tumour microenvironment [38] or from its beneficial effect on the tumour’s characteristic perineural invasion [39], as previously reported by our group [40]. Whilst Alliance 021501 closed early for futility in the SBRT arm, further research into the optimal radiation strategy to use in a NAT setting is warranted. Adaptive magnetic resonance guided radiotherapy (MRgRT) has been demonstrated to be feasible and safe in delivering dose-escalated radiation to a biologically effective dose ≥ 100 Gy. Ongoing trials are evaluating whether fractionated chemoradiation or SBRT is preferred as part of a TNT approach [41].

In summary, while BR and LA pancreatic cancer differ in anatomical staging at diagnosis, this study suggests that they may share comparable survival outcomes when treated with modern neoadjuvant strategies. The key distinguishing factor between groups was resectability rate and margin status rather than diagnostic staging or perioperative outcomes. With these results, the authors propose a treatment paradigm in which BR and LA patients are managed along a treatment continuum, with resectability determined dynamically by treatment response rather than fixed anatomical criteria at presentation. The difference in rates of resection between the BR and LA groups also highlights the ongoing challenge of rendering patients with LA disease to a state of resectability despite a total neoadjuvant approach.

### 4.1. Clinical Implications

Taken together, the authors suggest a two-tiered therapeutic strategy. First, all patients with BR/LA PDAC should receive multi-agent SACT, ideally FOLFIRINOX, as this regimen was strongly associated with higher rates of conversion to surgery. Second, for patients with stable or responsive disease but not resectable due to anatomical relationships, consolidation with CRT may prolong survival and improve local disease control. In surgical candidates, CRT following SACT in margin-threatened patients prior to surgery appears to be a safe treatment. In addition, surgical selection should be driven by response to neoadjuvant therapy rather than baseline radiological classification.

### 4.2. Strengths and Limitations

The strengths of this study include its relatively large cohort size, homogeneous treatment protocol within a high-volume UK tertiary centre over long-term follow-up, and modern relevance given the widespread adoption of FOLFIRINOX. However, several limitations should be acknowledged, primarily associated with the retrospective nature of the analysis. There is an imbalance in cohort size between the BR/LA groups, and smaller surgical subgroups analysis may be underpowered, increasing the risk of type II errors, and therefore negative findings should be interpreted with caution. With cohort studies, there is inherent selection bias between the treatment groups. Surgical patients selected after SACT +/− CRT were a self-selected cohort with better performance status, lower disease burden and proven less aggressive biology. Resected CRT patients would have also demonstrated better biology prior to resection, which in part may account for their superior survival. We also recognise that the analysis is susceptible to immortal time bias, as patients must survive before they undergo resection and future studies should include time-dependent methods to mitigate this bias. In addition, due to the unavailability of Gemcitabine plus nab-paclitaxel in BR/LA PDAC, we were unable to assess whether this regimen would have a similar or better outcome compared to FOLFIRINOX/GEM-CAP in our cohort. Despite these limitations, the observed consistency of survival differences with stepwise improvement across treatments suggests a strong association. These findings are hypothesis-generating and support further prospective evaluation of a TNT approach in selected BR/LA patients.

### 4.3. Future Directions

Our findings support ongoing international efforts to refine neoadjuvant strategies in PDAC. The potential role of radiation in conjunction with systemic treatment in a TNT approach for both BR and LA patients in optimising surgical outcomes and prolonging survival in non-resected patients warrants prospective evaluation in randomised clinical trials. We also await outcomes of phase III neoadjuvant clinical trials in resectable PDAC [42,43]. Future work should also focus on biomarkers of response to guide treatment sequencing, and on novel systemic agents such as RAS inhibitors to address the high rates of distant failure despite optimal local therapy. Circulating DNA may have a role in personalising treatment sequences [44]. Prediction of margin involvement (R1) remains a challenge; in the future, technologies including radiomics will play a role in this domain. There is also a clear need to redefine the definition of resectability, which should not be solely based on radiological findings but must also take into account tumour biology and the dynamic assessment of treatment response [45]. The type of neoadjuvant treatment may also influence radiographic and pathological interpretation following treatment [46], which needs to be further evaluated.

## 5. Conclusions

This single-centre UK series demonstrates that despite anatomical differences at diagnosis, borderline resectable and locally advanced pancreatic cancer may share comparable survival outcomes when treated with contemporary total neoadjuvant strategies. These findings challenge traditional radiological staging-based treatment paradigms and support a hypothesis in which a continuum-based approach, where resectability is dynamically defined by response to therapy. Ultimately, a margin-negative surgical resection offered the only chance of long-term survival for patients with BR/LA PDAC in our study.

## Figures and Tables

**Table 1:** Baseline characteristics of the study population comparing the BR and LA groups.

	BR	LA	*p* Value
*n*	44	121	
Age, median years (IQR)	70.0 (55.5–75.0)	68.0 (58.0–75.0)	0.925
BMI, median kg/m^2^ (IQR)	24.8 (22.9–27.5)	25.9 (21.0–27.3)	0.804
Female:male, *n* (%)	19 (43):25 (57)	58 (48):63 (52)	0.715
ECOG PS, *n* (%)			
0	20 (45.5)	43 (35.8)	0.533
1	20 (45.5)	64 (53.3)	
2	4 (9.1)	13 (10.8)	
T stage, *n* (%)			
T2	22 (50.0)	0 (0.0)	<0.001
T3	19 (43.2)	11 (9.1)	
T4	3 (6.8)	110 (90.9)	
N stage, *n* (%)			
N0	27 (61.4)	53 (43.8)	0.111
N1	16 (36.4)	60 (49.6)	
N2	1 (2.3)	8 (6.6)	
Ca19-9 at diagnosis, median U/mL (IQR)	299.0 (52.0–1272.5)	338.0 (48.0–1700.0)	0.995
SACT, *n* (%)			
FOLFIRINOX	29 (65.9)	69 (57.0)	0.470
Gemcitabine and Capecitabine (GEM-CAP)	14 (31.8)	38 (31.4)	
Gemcitabine	1 (2.3)	12 (9.9)	
Other	0 (0.0)	1 (0.8)	
SACT Cycles and Treatment Toxicity, *n* (%)			
FOLFIRINOX, median cycles (IQR)	7.0 (5.0–12.0)	11.0 (6.0–12.0)	0.185
Completed 12 cycles of FOLFIRINOX	19 (65.5)	36 (52.2)	0.357
FOLFIRINOX grade 3 toxicity	14 (48.3)	37 (53.6)	0.359
GEM-CAP, median cycles (IQR)	5.0 (4.0–6.0)	5.5 (3.0–6.0)	0.865
Completed 6 cycles of Gem-Cape	10 (71.4)	23 (60.5)	0.689
GEM-CAP grade 3 toxicity	1 (7.2)	3 (7.9)	1.0

**Table 2:** Univariate and multivariate analysis of factors that affect OS in this study.

Variable	Univariate			Multivariate		
	Comparison	HR (95% CI)	*p* Value	Comparison	HR (95% CI)	*p* Value
Age	(Per year)	1.01 (1.00–1.03)	0.098			
Sex	Male vs. female	1.01 (0.72–1.40)	0.972			
Performance status	(Per unit)	1.35 (1.04–1.74)	0.024	(Per unit)	1.28 (0.99–1.66)	0.060
BR/LA	LA vs. BR	1.33 (0.90–1.95)	0.148			
Initial Ca19-9	Per log10 unit	1.19 (1.03–1.38)	0.022	Per log10 unit	1.17 (1.00–1.39)	0.057
T-stage	T3 vs. T2	0.68 (0.37–1.26)	0.217			
	T4 vs. T2	1.11 (0.67–1.83)	0.686			
N-stage	N1 vs. N0	1.25 (0.89–1.74)	0.192	N1 vs. N0	0.89 (0.62–1.27)	0.525
	N2 vs. N0	2.24 (1.07–4.69)	0.032	N2 vs. N0	0.72 (0.33–1.59)	0.420
SACT type	Gem-Cape vs. FOLFIRINOX	1.98 (1.37–2.86)	<0.001	Gem-Cape vs. FOLFIRINOX	1.10 (0.74–1.62)	0.648
	Gem vs. FOLFIRINOX	2.46 (1.35–4.47)	0.003	Gem vs. FOLFIRINOX	0.66 (0.33–1.32)	0.242
	Other vs. FOLFIRINOX	0.66 (0.16–2.67)	0.555	Other vs. FOLFIRINOX	0.36 (0.09–1.53)	0.167
CRT	CRT vs. none	0.42 (0.30–0.58)	<0.001	CRT vs. none	0.27 (0.18–0.40)	<0.001
Surgery	Surgery vs. none	0.22 (0.14–0.35)	<0.001	Surgery vs. none	0.15 (0.09–0.27)	<0.001

**Table 3:** Baseline characteristics and surgical outcomes of resected patients.

	BR	LA	*p* Value
*n*, (%)	17	18	
Age, median years (IQR)	67.0 (58.0–71.0)	61.0 (56.0–69.5)	0.390
BMI, median kg/m^2^ (IQR)	24.0 (21.0–26.0)	21.0 (19.25–26.75)	0.456
Female:male, *n* (%)	7 (41):10 (59)	9 (50):9 (50)	0.854
ECOG PS, *n* (%)			
0	11 (64.7)	7 (38.9)	0.533
1	6 (35.5)	11 (61.1)	
2	0 (0.0)	0 (0.0)	
T Stage at Diagnosis, *n* (%)			
T2	9 (52.9)	0 (0.0)	<0.001
T3	8 (47.1)	3 (16.7)	
T4	0 (0.0)	15 (83.3)	
N stage at diagnosis, *n* (%)			
N0	15 (88.2)	7 (38.9)	0.008
N1	2 (11.8)	11 (61.1)	
N2	0 (0.0)	0 (0.0)	
Ca19-9 at diagnosis, Median U/mL (IQR)	298.0 (83.5–936.5)	310 (12.25–1396.25)	0.777
SACT, *n* (%)			
FOLFIRINOX	16 (94.1)	15 (83.3)	0.512
Gemcitabine and Capecitabine (GEM-CAP)	1 (5.9)	2 (11.1)	
Other	0 (0.0)	1 (5.6)	
Cycles *Median cycles (IQR)*	10.0 (6.0–12.0)	11.0 (8.0–12.0)	0.512
Completed planned SACT	8 (47.1)	9 (50.0)	1.0
Grade 3 SACT toxicity	8 (47.1)	12 (66.7)	0.394
CRT	8 (47.1)	13 (72.2)	0.241
Operation, *n* (%)			
Pancreaticoduodenectomy	14 (82.3)	14 (77.8)	0.710
Distal pancreatectomy and splenectomy	1 (5.9)	1 (5.6)	
Total pancreatectomy	2 (11.8)	2 (11.1)	
Other	0 (0.0)	1 (5.6)	
Histological T Staging, *n* (%)			
ypT4	0 (0.0)	1 (5.6)	0.648
ypT3	1 (5.9)	1 (5.6)	
ypT2	8 (47.1)	5 (27.8)	
ypT1	4 (23.5)	7 (38.9)	
ypT0	4 (23.5)	4 (22.2)	
Histological N Staging, *n* (%)			
ypN1	4 (23.5)	4 (22.2)	1.0
ypN0	13 (76.5)	14 (77.8)	
Operative Outcomes, *n* (%)			
Venous resection	8 (47.1)	8 (44.4)	1.0
R1	5 (29.4)	6 (33.3)	1.0
Postoperative pancreatic fistula Grade A	1 (5.9)	1 (5.5)	0.959
Postoperative pancreatic fistula Grade B	1 (5.9)	0 (0.0)	
Delayed gastric emptying	2 (11.8)	3 (16.7)	1.0
Clavien–Dindo 3+	3 (17.6)	4 (22.2)	1.0
30-day readmission	5 (29.4)	3 (16.7)	0.62
Adjuvant treatment	3 (17.6)	4 (22.2)	1.0

**Table 4:** Summary of log rank test and univariate Cox HR (95% CI) in the surgical cohort with respective stratifications.

Groups	Median OS (Months)	Log Rank X^2^	*p* Value	Univariate Cox HR (95% CI)	*p* Value
BR v LA	45 v 34	0.55	0.458	1.37 (0.58–3.24)	0.467
R1 v R0	22 v 47	14.34	0.00015	4.50 (1.91–10.61)	<0.001
SACT and CRT v SACT	33 v 22	1.89	0.168	0.56 (0.24–1.31)	0.181
Surgery R1 vs. SACT and CRT (non-surgical)	22 v 20	1.12	0.289	0.70 (0.36–1.38)	0.306

**Table 5:** Summary of recurrence in the surgical cohort with respective stratifications.

	BR	LA	*p*
Recurrence	11 (64.7)	12 (66.7)	1.0
Distribution			
Locoregional	0 (0.0)	5 (41.7)	0.031
Distant	5 (45.5)	5 (41.7)	
Both	6 (54.5)	2 (16.7)	
Time to recurrence, median months (IQR)	9.0 (7.0–10.5)	9.0 (4.0–13.5)	0.692
Palliative treatment	7 (41.2)	6 (33.3)	0.897
	SACT	CRT	
Recurrence	12 (85.7)	11 (52.4)	0.095
Distribution			
Locoregional	2 (16.7)	3 (27.3)	0.720
Distant	5 (45.5)	5 (45.5)	
Both	3 (27.3)	3 (27.3)	
Time to recurrence, median months (IQR)	9.5 (6.5–14.3)	8.5 (6.0–9.0)	0.371
Palliative treatment	6 (42.9)	7 (33.3)	0.830
	R0	R1	
Recurrence	13 (54.1)	10 (90.9)	0.055
Distribution			
Locoregional	4 (30.7)	1 (10.0)	0.309
Distant	4 (30.7)	6 (60.0)	
Both	5 (38.5)	3 (30.0)	
Time to recurrence, median months (IQR)	9.0 (7.0–13.5)	7.0 (5.2–10.5)	0.208
Palliative treatment	8 (61.5)	5 (50.0)	0.708

## Data Availability

Data presented are contained within the article. The authors can be contacted for data requests; requests will only be considered for academic collaboration purposes.

## Supplementary Materials

The following supporting information can be downloaded at: https://www.mdpi.com/article/10.3390/cancers18101597/s1, Table S1: Surgical outcomes comparing resected patients treated with neoadjuvant SACT and CRT.
