# Supplementary material for: Total Neoadjuvant Approach for Borderline Resectable and Locally Advanced Pancreatic Adenocarcinoma—UK Tertiary Cancer Centre Experience
Source: Cancers (Basel). 2026 May 14;18(10):1597. doi: 10.3390/cancers18101597 (PMC13205041; doi:10.3390/cancers18101597)
Supplement: Supplementary file 1 [file cancers-18-01597-s001.zip › cancers-4259174-supplementary.pdf]

Supplementary

Supplementary Table S1. Surgical outcomes comparing resected patients treated with neoadjuvant SACT and CRT

n(%)	SACT	CRT	p
**Venous Resection**	8 (57.1)	8 (38.1)	0.45
**R1**	8 (38.1)	3 (21.4)	0.50
**Post Operative Pancreatic Fistula Grade A**	0 (0.0)	2 (9.5)	1.0
**Post Operative Pancreatic Fistula Grade B**	1 (7.1)	0 (0.0)	
**Delayed Gastric Emptying**	2 (14.3)	3 (14.3)	1.0
**Clavien Dindo 3+**	2 (14.3)	5 (23.8)	0.80
**30-day Readmission**	4 (28.6)	4 (19.0)	0.81
**Adjuvant Treatment**	5 (35.6)	2 (9.5)	0.14
